# Postoperative Pain Due to a Retained Needle in the Mesentery of the Ascending Colon: A Case Report

**DOI:** 10.7759/cureus.77952

**Published:** 2025-01-25

**Authors:** Michail Angelos Papaoikonomou, Europi Michailidou, Aggeliki Chlorou, Nikolaos Krokos

**Affiliations:** 1 Department of General Surgery, Agios Pavlos General Hospital, Thessaloniki, GRC

**Keywords:** acute postoperative pain, foreign body, mesentery, migration, needle

## Abstract

A plethora of reports have previously been published concerning the removal of intra-abdominal foreign bodies. However, in most of these cases, an acute inflammatory reaction occurred. In emergency medicine, accidental sewing needle ingestion is a frequent presentation. However, it is extremely uncommon for the needle to be inserted through the abdominal wall and remain silent inside the abdominal cavity. We hereby report a case of incidental finding of a needle retrieved from the antimesenteric intestinal border of the ascending colon in a 41-year-old female patient who presented to our hospital with acute right abdominal colic on the third postoperative day after laparoscopic cholecystectomy. Laparotomy was performed, and the corroded needle was successfully removed from the mesentery together with its sheath.

## Introduction

The presence of sewing needles has been documented in various anatomical locations, including the gastrointestinal tract, cranium, and heart. As demonstrated in the extant literature, numerous reports of intraperitoneal foreign bodies have documented a broad spectrum of presentations [1-4]. The entry of foreign bodies into the body cavities can occur through the ingestion process and a penetrating injury [5]. The occurrence of foreign body penetrations can be attributed to three distinct categories: accidental, self-inflicted, and physical abuse [1,3,5]. In the initial assessment of the case, no patient-related cause was identified. However, upon consideration of the patient's past medical history, the possibility of penetrating injury was suspected. The assumption was made based on the exclusion of other possible mechanisms of foreign body retention, including foreign body ingestion, physical abuse/torture, or self-destructive behavior. The present study details the case of a sewing needle found in the mesentery of the ascending colon in a female patient who experienced exacerbating colic in the right abdomen after laparoscopic cholecystectomy attributed to an intra-abdominal extraluminal sharp foreign body. Even though laparoscopic exploration is generally preferred, laparotomy was preferred for the management of this patient due to safety reasons.

## Case presentation

A 41-year-old female patient had a laparoscopic cholecystectomy for cholelithiasis discovered by ultrasound before surgery. The patient had been suffering from sporadic episodes of discomfort in the right upper quadrant for months. The patient's BMI was 22 kg/m2; she was currently unemployed and residing in a rural area. She had given birth to a single child naturally and had no past medical history or mental illness. Furthermore, she had not undergone any surgical operations before this. Professional surgical standards were followed during the procedure, and the "critical view of safety," which is employed in laparoscopic cholecystectomy to identify the cystic duct before transection clearly, was attained. On the first postoperative day, the patient was released from the hospital in good overall health. The patient was brought to the surgical unit for additional assessment of her condition after presenting to our hospital on the third postoperative day with severe colic pain in the right upper quadrant radiating to the right iliac fossa.

**Figure 1:**
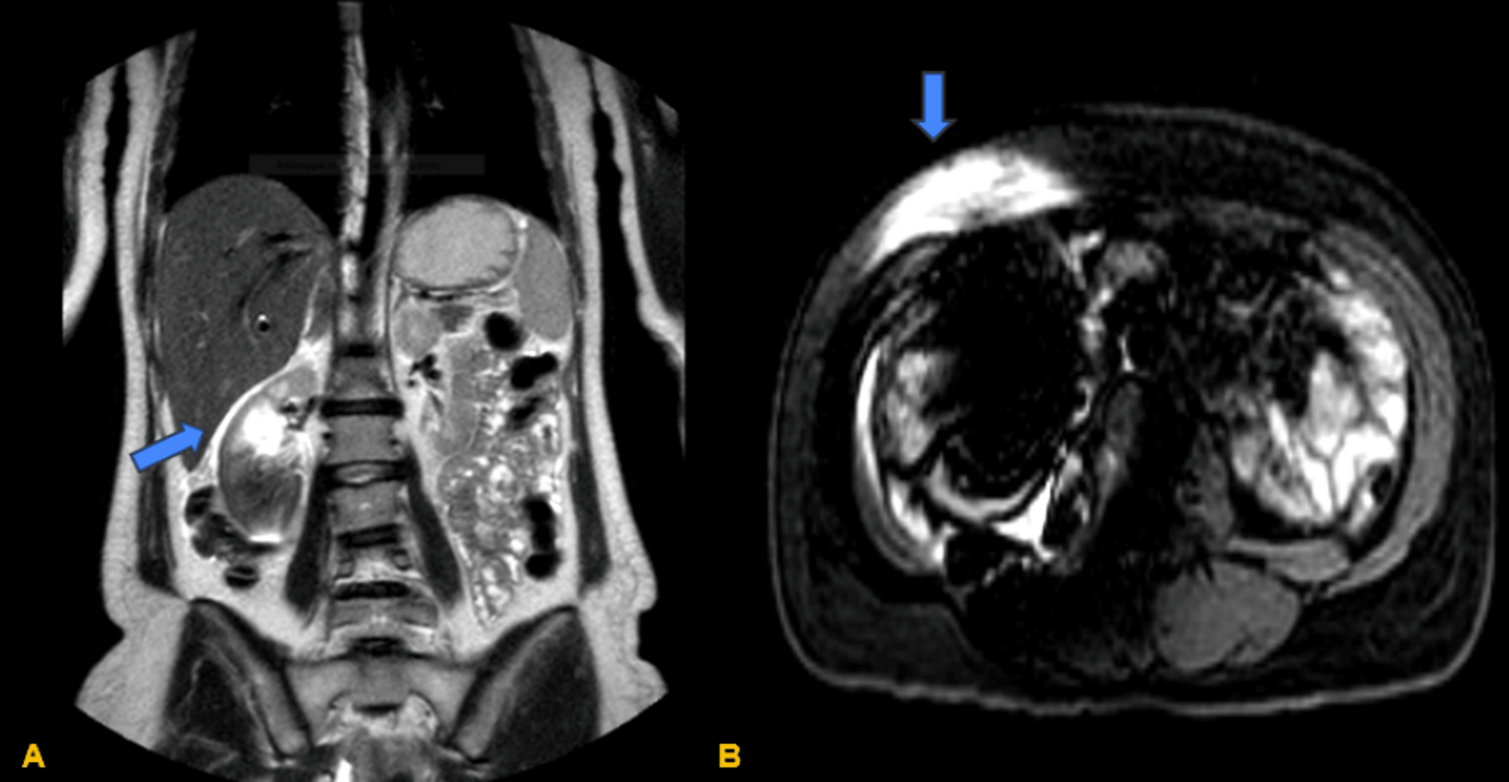
MRI scan of the abdomen. (A) Coronal view of the abdomen showing an artifact (blue arrow) in the right abdomen on the T2 sequence. (B) Transverse view of the artifact (blue arrow) caused by a metallic object discovered during the procedure on the T1 sequence

On arrival, the patient was fully alert and oriented. Her blood pressure was 130/90 mmHg, her temperature was 36.6 °C, her pulse rate was 85/minute, and her respiration rate was 18/minute while her physical examination revealed abdominal rigidity on the right hypochondrium with radiation to the right iliac fossa. Her laboratory parameters were WBC 9.5 x 10^3^/μL with neutrophils 77.1%, Hb 11 g/dL, hematocrit 32.3%, C-reactive protein 2.96 mg/dL, serum glutamic-oxaloacetic transaminase 16 international units (IU)/L, serum glutamic-pyruvic transaminase 45 IU/L, total bilirubin 1.15 mg/dL, direct bilirubin 0.49 mg/dL, gamma-glutamyl transferase 54 IU/L, and alkaline phosphatase 114 IU/L. Our differential diagnosis included postoperative fluid collection or hematoma, choledocholithiasis, and renal colic pain. An upper abdominal ultrasound was ordered, which showed no fluid collection or biliary dilatation. No focal liver lesion was noted, while the common bile duct, spleen, and pancreas appeared normal. A urological assessment was also carried out to rule out any urological diagnosis that could be related to the patient's symptoms, and both kidneys appeared normal on ultrasound, with no evidence of kidney stones or hydronephrosis. The surgical team recommended a magnetic resonance cholangiopancreatography to evaluate the biliary system in detail, which was scheduled for the following day. However, the procedure was subsequently halted due to the presence of a large artifact on the right abdomen, and only T1- and T2-weighted images were obtained (Figure 1).

**Figure 2:**
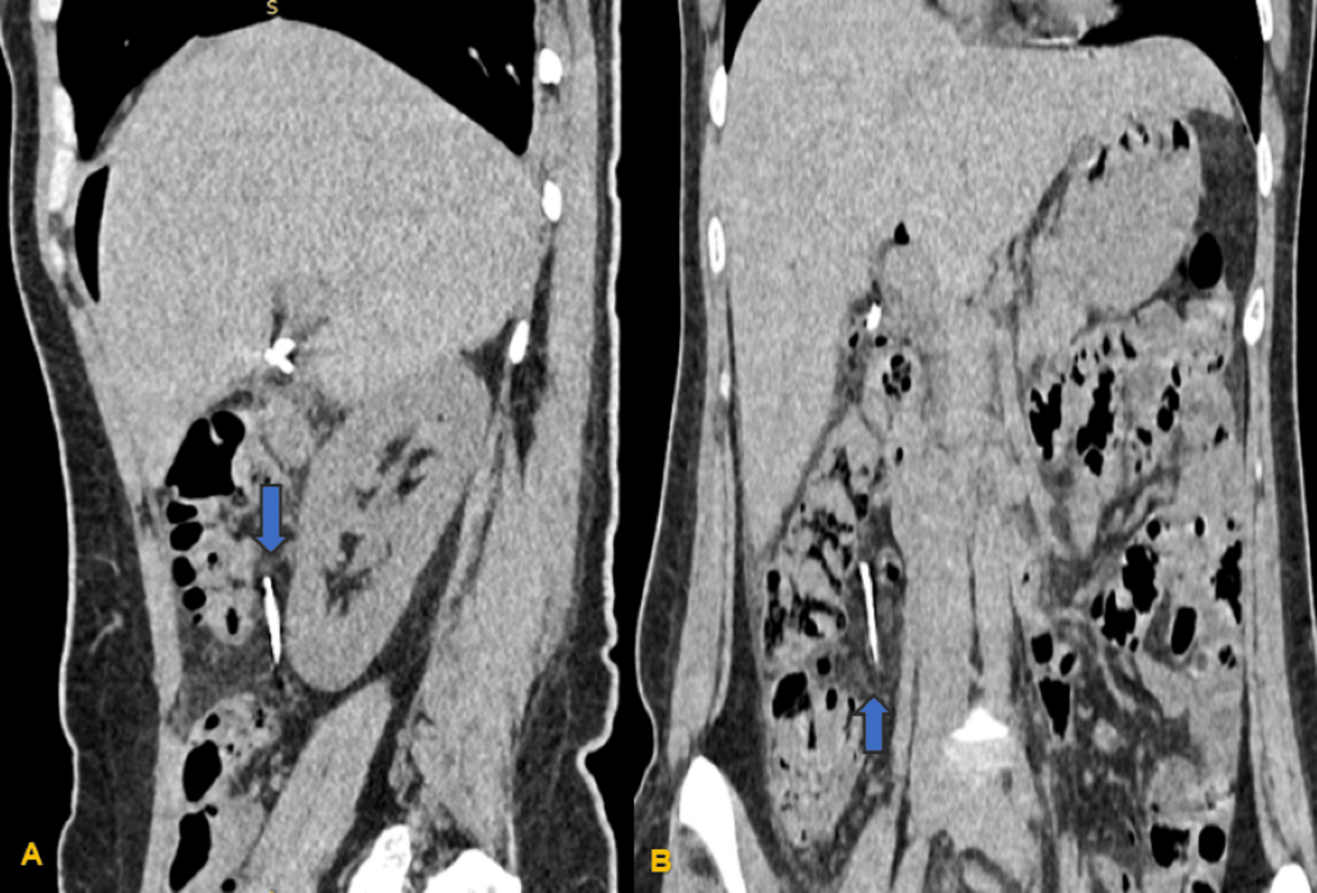
(A) Sagittal plane showing the presence of a longitudinal sharp object (blue arrow) in front of the lower pole of the right kidney located posterior to the ascending colon. (B) Coronal plane of the CT abdomen showing the presence of a sharp foreign body (blue arrow) inferior to the umbilicus, 6-7 cm to the right of the medial line and 3 cm below the right rectus abdominis muscle

The radiologist proceeded with the evaluation with a CT scan, which revealed a thin, longitudinal metallic foreign body measuring 3.5 cm with vertical orientation. The foreign body was located in the posterior mesentery of the ascending colon and anterior to the lower pole of the right kidney. The rest of the CT scan findings were unremarkable, with the exception of minor fluid accumulation in the lesser pelvis, a minor right pleural effusion, and focal free air within the abdominal cavity. These are attributable to the recent laparoscopic cholecystectomy performed on the patient. Both intrahepatic and extrahepatic bile ducts showed no dilatation (Figure 2).

**Figure 3:**
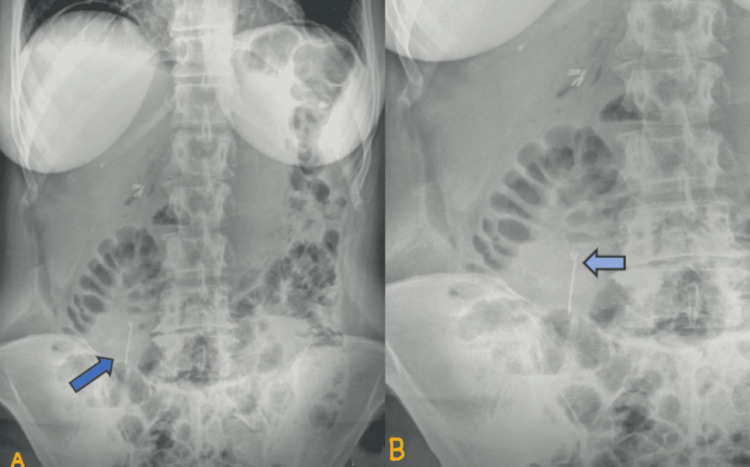
(A) Abdominal X-ray showing the foreign object (blue arrow) that was detected on the CT scan and exhibiting the futures of a sewing needle. (B) Closer view of the abdominal X-ray pointing out the sheath of the needle (light blue arrow)

Subsequently, the patient underwent an erect abdominal X-ray to facilitate the nature of the foreign object, which was found to have the features of a sewing needle (Figure 3).

**Figure 4:**
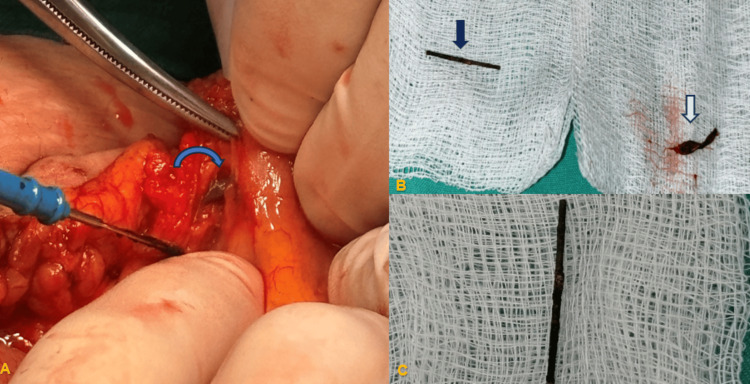
(A) Surgical field where the sewing needle (blue arrow) was found in the mesentery of the ascending colon. (B) The sewing needle (dark blue arrow) and its sheath (white arrow). (C) Closer view of the corroded sewing needle that was retained

To further clarify the origin of the needle in the patient's abdomen, it was ascertained that no prior incidents were disclosed. This included no cases of needle penetration, ingestion, or any professional connection to sewing. The possibility of physical abuse/torture or self-inflicted injury with sewing needles was also excluded. The surgical team decided to carry out an exploratory laparotomy to remove the foreign object without a laparoscopic exploration effectively. A small supraumbilical medial incision was made, and the team successfully entered the abdominal cavity, where no adhesions were present. During the exploratory laparotomy, the large intestine was checked, and a fragile sewing needle encased in its adjacent fibrinous reaction was palpated in the antimesenteric side of the mesentery of the ascending colon, in proximity to the intestinal wall without penetrating the lumen (Figure 4A). The foreign body with its fibrinous enclosure was safely removed, leaving the ascending colon intact (Figures 4B, 4C).

The patient was permitted to commence a clear fluid diet immediately and received intravenous broad-spectrum antibiotics (cefuroxime 750 mg, three times a day) for two days. Thereafter, the patient exhibited a normal postoperative course and was discharged on the third postoperative day.

## Discussion

Foreign bodies may reach the intraperitoneal space via the mouth, anus, urogenital tract, or percutaneously [6]. The retainment of foreign bodies in the abdomen, such as needles, is most frequently observed in children, which is related to accidental digestion [6]. Although rare in adults, there is a higher prevalence among individuals with mental disabilities, prisoners, and young girls who wear turbans in Islamic countries [7]. The overall complication rate from foreign body ingestion is estimated to be less than 1% [4,8]. All sharp foreign bodies must be meticulously removed before passing through the gastrointestinal tract, as there is a documented prevalence of 15%-35% of such foreign bodies perforating the intestinal wall, typically in the area of the ileocecal valve [4,9]. Foreign bodies passing through the gastrointestinal tract are usually spontaneous and uncomplicated, presenting with a range of symptoms from asymptomatic to acute abdominal pain, perforation, and peritonitis [8]. Sharp objects can also cause erosions and bleeding [4,9]. The presence of noningested intraperitoneal foreign bodies has been demonstrated to result in chronic abdominal pain [1,3]. The majority of patients are unable to recall any instances of unintentionally swallowing or penetrating foreign bodies, as in our case. Consequently, the diagnosis in such patients poses a significant challenge [2,3]. The presentation of symptoms can be nonspecific and may resemble those of other conditions, as observed in this case. Standing X-rays are widely utilized to diagnose foreign bodies in the abdominal area; however, they may not provide exact localization as effectively as abdominal CT scans [2,9]. In this case, the patient presented with acute abdominal pain in the upper right quadrant of the abdomen following the laparoscopic cholecystectomy. As a result, the possibility of a foreign body was not included in the initial diagnosis. Abdominal CT plays an important role in the diagnosis, localization, and determination of the surgical procedure in complicated cases [2]. The laparoscopic surgical approach is the most common method of abdominal foreign body extraction in stable patients without signs of acute abdomen due to its numerous advantages, including reduced postoperative pain, decreased incidence of wound infection, and minimal surgical stress [2,3]. Especially in cases where the retained foreign body is located in proximity to the intestinal wall, as in this case, the open approach is advocated. The surgical team's decision to adopt the open approach was driven by the necessity to ensure optimal access to the antimesenteric border of the ascending colon, thereby enhancing patient safety. Additionally, if the patient had been presented with an abdominal X-ray during the initial assessment and the presence of the sewing needle had been known preoperatively, our approach might have been open, allowing for the management of cholelithiasis and foreign body retention in a single surgical intervention. The location significantly impacts the prognosis and whether or not the item can be reached via endoscopy [6]. To enhance the prognosis of foreign bodies lodging in rare sites, early detection and prompt treatment are crucial, as missed or delayed diagnosis has been linked to a mortality rate of up to 10% [8].

## Conclusions

In conclusion, we encountered a case of postoperative pain after laparoscopic cholecystectomy where we managed to detect a sewing needle from a patient's abdominal cavity as a cause of it. It was supposed that years ago, it might have entered the abdominal cavity and stayed there silently without causing any inflammation. Due to the formation of pneumoperitoneum during laparoscopic cholecystectomy, we presume that the foreign body must have slightly moved closer to the intestinal wall, irritating the ascending colon. Given that her discomfort was colic-like and followed the large intestine's natural peristalsis, this might help to explain its nature. The likelihood of a foreign body being identified as the underlying cause of postoperative pain following laparoscopic cholecystectomy is low. However, once a comprehensive and detailed medical history has been obtained, the possibility should be considered. This case report highlights the significance of meticulous medical history-taking and adequate preoperative evaluation, while also delineating the management of cases that transform from elective to perplexing.
